# Toxic stress on a pediatric population during the COVID-19 pandemic

**DOI:** 10.1590/1984-0462/2023/41/2021399

**Published:** 2023-03-13

**Authors:** Anna Beatriz Galheiro Silvério, Denise Leal de Souza, Gabriela de Sio Puetter Kuzma, Gabriela Storithont Mudri, Isabelle Beatriz Freccia Nagel, Julia Concer da Silva, Leonardo Cecconello, Raissa Peres de Oliveira, Raissa Wurth Marchinhacki, Samantha Nagasako Soejima

**Affiliations:** a Fundação Universidade Regional de Blumenau, Blumenau, SC, Brazil.

**Keywords:** COVID-19, Pandemic, Toxic stress, Child, Adolescents, COVID-19, Pandemia, Estresse tóxico, Crianças, Adolescentes

## Abstract

**Objective:**

The aim of this study was to analyze the impact of social isolation as a result of the coronavirus-19 disease (COVID-19) pandemic in children and adolescents aged 0–17 years in Southern Brazil.

**Methods:**

This is a cross-sectional study in which 542 questionnaires answered by parents or legal guardians of children and adolescents aged 0–17 years residing in the Brazilian South region, through Google Forms^®^. Questionnaires answered incompletely or from children outside the stipulated age group and from other regions of the country were excluded from the research. The collected data were organized into descriptive and association tables containing absolute and relative frequencies, medians, averages, standard deviations, quartile deviations, average, and proportion estimates in the form of 95% confidence intervals and the result of the chi-square test of independence. Data analysis was fulfilled with the application aid of Microsoft Excel 2016 and Epi Info version 7.2.1.0 of 01/27/2017. Statistically significant associations were considered when p<0.05.

**Results:**

There was an increase in the perception of nervousness (62.7%), anxiety (67.7%), and sadness (51.3%) in children and adolescents during the pandemic period. This study evidenced a high prevalence of screens overuse (50.9%) and sedentary lifestyle (39.1%) in this age group during the period. Furthermore, the occurrence of regressive behaviors occurred more frequently in the age group of 3–6 years (57.1%) and between 7 and 10 years (44.6%).

**Conclusions:**

It is inferred from this study that one of the implications resulting from the pandemic period is the increase of toxic stress in the pediatric population.

## RESUMO

**Objetivo:**

Analisar o impacto do isolamento social em decorrência da pandemia associada ao coronavírus 2019 (COVID-19), em crianças e adolescentes de zero a 17 anos da Região Sul do Brasil.

**Métodos:**

Trata-se de um estudo de corte transversal em que foram obtidos 542 questionários, respondidos por pais ou responsáveis legais de crianças e adolescentes de zero a 17 anos residentes da Região Sul brasileira, por meio do Google Forms^®^. Questionários respondidos de maneira incompleta ou de crianças fora da faixa etária estipulada e de outras regiões do país foram excluídos da pesquisa. Os dados colhidos foram organizados em tabelas descritivas e de associação contendo frequências absolutas, relativas, médias, medianas, desvios padrão, desvios quartílicos, estimativas de média e de proporção em forma de intervalos com 95% de confiança e resultado do teste do qui-quadrado de independência. A análise dos dados foi realizada com o auxílio dos aplicativos Microsoft Excel 2016 e Epi Info versão 7.2.1.0, de 27 de janeiro de 2017. Foram consideradas associações estatisticamente significativas quando p<0,05.

**Resultados:**

Houve aumento da percepção de nervosismo (62,7%), ansiedade (67,7%) e tristeza (51,3%) em crianças e adolescentes no período da pandemia. O estudo evidenciou alta prevalência de uso excessivo de telas (48,2%) e de sedentarismo (39,1%) nessa faixa etária durante o período. Ademais, a ocorrência de comportamentos regressivos ocorreu de maneira mais frequente nas faixas etárias de 3–6 anos (57,1%) e de 7–10 anos (44,6%).

**Conclusões:**

Infere-se deste estudo que uma das implicações do período da pandemia é o aumento do estresse tóxico na população pediátrica.

## INTRODUCTION

Pandemics do not affect the world quite often; thus, when they break out, they affect the lives of children and adults in a devastating way.^1^ Although children have milder forms of coronavirus-19 disease (COVID-19), they can be more affected in terms of psychological development for being a vulnerable population.^2^

According to the World Health Organization (WHO), the closing of educational institutions as an initiative to contain cases of COVID-19 took away about 1.5 billion children and adolescents from schools, a measure that also affected Brazil.^3^ Consequently, this period requires a change in family dynamics, with parents/responsible persons needing to reconcile remote work with children’s activities.^4^ At the social level of living, an increase in stress within this domestic sphere was noted, often resulting in situations of violence since the proximity to abusive parental figures may result in punishment practices, both physically and psychologically.^3,4^

Furthermore, the pandemic resulted in an environment predisposed to the development of toxic stress.^4^ This type of stress represents a strong and extensive sensation of physical and emotional symptoms, such as tiredness and depression, and signs of aggression and restlessness. This effect triggers high levels of cortisol in the body of young people, promoting changes in the immune and nervous systems which are responsible for emotion, memory, and learning.^2^

Therefore, the social isolation resulting from the COVID-19 pandemic brought many effects to the population as a whole; however, the emphasis given to young people demonstrates that this group is going through a period of consequences not only social but biologicals too, affecting the coexistence with other individuals as well as psychological health.^3^ In the meantime, a study was carried out with the objective of analyzing the impact of social isolation on children and adolescents in Southern Brazil.

## METHOD

A cross-sectional study was carried out. Questionnaires collected through the Google Forms^®^ platform were sent randomly in a similar number between public and private schools through the WhatsApp Application to the responsible persons of children and adolescents aged between 0 and 17 years. Considering a questionnaire was illed out for each minor, in case if the responsible persons had more than one child, questionnaires were answered according to the number of children. Data were collected between November 2020 and January 2021, during the COVID-19 pandemic.

The sample size calculation was performed with 95% confidence level for an estimated population of children and adolescents of 2,300,000 according to the 2010 census of the IBGE (Brazilian Institute of Geography and Statistics).^5^ This calculation resulted in 385 children/adolescents needed in this study. Inclusion criteria were being parents or guardians of children and adolescents aged 0–17 years living in the Southern Region of Brazil. Exclusion criteria were as follows: residence outside the region of this study, incomplete questionnaires, or questionnaires outside the studied age group.

The database was built in the Microsoft Office Excel 2016 and for statistical analysis the Epi Info version 7.2.1.0 of 01/27/2017 was used.

Data were organized in descriptive and association tables containing median, average, relative and absolute frequencies, standard deviations, quartile deviations, average and proportion estimates in form of intervals with 95% confidence, and the result of the chi-square test of independence. Statistically significant associations were considered when p<0.05.

The variables analyzed were as follows: number of siblings, age, and gender of the child or adolescent; relationship of the minor with the responsible person who is answering the questionnaire; educational level of the responsible person; monthly family income; number of people in the residence; time and level of social isolation of the responsible person; level of social isolation of the child or adolescent; psychic symptoms and sleep quality of the responsible person and the child or adolescent; presence of conflict between family members; practice of physical activity, use of electronic devices, and school activities of the child or adolescent; regressive symptoms; and suspicion of COVID-19 in a family member or death of an acquaintance of the family due to COVID-19. The psychic symptoms evaluated were tiredness, nervousness, anxiety, sadness, and aggressiveness.

The association between psychological symptoms and age, psychological symptoms and exposure to electronic devices, between regressive behaviors and age and regressive behaviors and sex were analyzed.

There was no identification on the form of the responsible person or the child, ensuring their anonymity. Therefore, the need of the Free and Informed Consent Form was dispensed.

This study was approved by the Research Ethics Committee of the Blumenau Regional University Foundation under protocol CAAE: 39364620.5.0000.5370. The questionnaire used is attached at the end of the work.

## RESULTS

A total of 677 questionnaires were obtained. The questionnaires from residents of other regions of the country, outside the Southern Region of Brazil, were excluded (n=111, as well as 24 incomplete questionnaires or questionnaires outside the studied age group studied). A total of 542 valid questionnaires were included, corresponding to a margin of error of 4.2%.

Table 1 presents a descriptive statistics in the form of absolute and relative frequency distributions and interval estimates with a 95% confidence level. There is a predominance of parents/responsible person with only one child (47.8%), male (52.0%) and aged between 3 and 6 years (33.9%). The questions were mostly answered by individuals who live in the State of Santa Catarina (77.1%) with a College degree (79.9%). In total, 73.8% of the participants do not live with people from the COVID-19 risk group. Most families in this study, at the time of data collection, were in partial social isolation (71.8%). The other epidemiological data can be seen in Table 1.

**Table 1.** Profile of respondents (n=542)

Variables	n (%)	95% confidence interval
1. How many children do you have between 0 and 17 years old?		
One	259 (47.8)	43.58–51.99
Two	242 (44.6)	40.46–48.83
Three or more	41 (7.6)	5.34–9.79
2. How old is your child? (years)		
0–2	67 (12.4)	9.19–15.13
3–6	184 (33.9)	29.96–37.93
7–10	166 (30.0)	26.75–34.51
11–17	125 (23.0)	19.52–26.61
3. What is your child’s gender?		
Male	282 (52.0)	47.82–56.24
Female	243 (44.8)	40.65–49.02
Not informed	17 (3.1)	0.67–4.60
4. What is your relationship with the child/adolescent?		
Biological mother	488 (90.0)	87.52–92.56
Biological father	41 (7.6)	5.34–9.79
Adoptive mother	5 (0.9)	0.12–1.73
Other	8 (1.5)	0.46–2.49
5. What is your education level?		
Elementary school incomplete or complete	20 (3.7)	2.10–5.28
High school complete or college incomplete	89 (16.4)	13.30–19.54
College complete	433 (79.9)	76.51–83.26
6. What is the average monthly income of your family?		
Up to R$ 5.000	152 (28.0)	24.26–31.83
From R$ 5.000 to R$ 10.000	133 (24.5)	20.92–28.16
Over R$ 10.000	257 (47.4)	43.21–51.62
7. How many people, including yourself, live in your house?		
Up to 3 people	250 (46.1)	41.93–50.32
4 or 5 people	283 (52.2)	48.01–56.42
6 people or more	9 (1.7)	0.58–2.74
8. How long have you been in social isolation?		
Not in isolation or up to 3 months	274 (50.5)	46.34–54.76
More than 3 months	268 (49.4)	45.24–53.66
9. What is your level of social isolation?		
None	144 (26.6)	22.85–30.29
Partial	389 (71.8)	67.98–75.56
Complete	9 (1.7)	0.58–2.74
10. What is the level of social isolation of people aged 0–17 years in your house?		
None	78 (14.4)	11.44–17.35
Partial	393 (72.5)	68.75–76.27
Complete	71 (13.1)	10.26–15.94

When asked if there was a suspicion of COVID-19 in any member of the house, 366 (67.5%) answered “No”, and 38.7% answered that they had known someone who died as a result of complications from this disease. When the responsible persons were asked about themselves, most reported being tired (81.0%), nervous (77.9%), anxious (79.3%), depressed (51.5%), and stressed (78.2%) compared to the period before the pandemic. When asked about sleep, 43.3% of the responsible persons reported having more difficulty in sleeping.

Regarding the children’s psychological symptoms, the responsible persons reported an increase in the perception of nervousness (62.7%), anxiety (67.7%), and sadness (51.3%) during the pandemic period (Table 2). Of the total, 38% reported missing medical appointment, vaccine, exam, or routine procedure due to the pandemic, and 35.1% had symptoms of anxiety when approached about issues related to COVID-19.

**Table 2.** Association between the occurrence of psychological symptoms and the child’s age.

How is your child currently doing compared to before the pandemic?	How old is your child?					p-value
	0–2 years (n=67; 12.4%)	3–6 years (n=184; 33.9%)	7–10 years (n=166; 30.6%)	11–17 years (n=125; 23.1%)	Total (n=542; 100%)	
A. Tired						
Same or less	57 (85.1)	101 (54.9)	88 (53.0)	66 (52.8)	312 (57.6)	<0.001
More	10 (14.9)	83 (45.1)	78 (47.0)	59 (47.2)	230 (42.4)	
B. Nervous						
Same or less	41 (61.2)	51 (27.7)	51 (30.7)	59 (47.2)	202 (37.3)	<0.001
More	26 (38.8)	133 (72.3)	115 (69.3)	66 (52.8)	340 (62.7)	
C. Anxious						
Same or less	46 (68.7)	46 (25.0)	39 (23.5)	44 (35.2)	175 (32.3)	<0.001
More	21 (31.3)	138 (75.0)	127 (76.5)	81 (64.8)	367 (67.7)	
D. Sad						
Same or less	52 (77.6)	81 (44.0)	69 (41.6)	62 (49.6)	264 (48.7)	<0.001
More	15 (22.4)	103 (56.0)	97 (58.4)	63 (50.4)	278 (51.3)	
E. Aggressive						
Same or less	47 (70.1)	87 (47.3)	67 (40.4)	88 (70.4)	289 (53.3)	<0.001
More	20 (29.9)	97 (52.7)	99 (59.6)	37 (29.6)	253 (46.7)	
F. Family conflicts						
Same or less	53 (79.1)	95 (51.6)	70 (42.2)	93 (74.4)	311 (57.4)	<0.001
More	14 (20.9)	89 (48.4)	96 (57.8)	32 (25.6)	231 (42.6)	

Large portion (39.1%) of the population reported that their children do not have physical activity and spend more than 4 h a day using electronic devices (48.2%). Regarding school activities, 75.5% of the responsible persons reported that their children attended virtual classes associated with performing tasks. The responsible persons reported that 35.1% of the children, when a conversation about COVID-19 was started, manifested some physical symptom (headache, epigastric pain, nausea, or dyspnea with no apparent explanation), irritability, or avoid this kind of conversation. As for the quality of sleep of their children, 63.1% of the responsible persons rated it as adequate.

When compared to the period before the pandemic, 231 (42.6%) reported more family conflicts; of which, 92 (39.8%) have got only one child; 116 (50.2%) have got two children aged between 0 and 17 years; and 23 (9.9%) have got three children aged between 0 and 17 years. There was a statistically significant association between age and the perception of tiredness, nervousness, anxiety, sadness, aggression, and in the frequency of family conflicts. The results are described in Table 2. There was no statistically significant association between the occurrence of these symptoms and the children’s gender.

Regarding screen time, we obtained 513 (94.6%) responses. Of these, half of the children (n=261; 50.9%) used the screen for more than 4 h a day. A statistical association was found when correlating the occurrence of psychic symptoms and the presence of family conflicts with the time using electronic devices for more than 4 h a day. These data are described in Table 3.

**Table 3.** Association between the occurrence of psychological symptoms and daily screen time.

How is your child currently doing compared to before the pandemic?	How much time per day is your child using electronic devices (television, tablet, computer, cell phone, and video game)? (Not counting virtual class period)				p-value
	Up to 2 h (113; 22.0%)	From 2 to 4 h (139; 27.1%)	More than 4 h (261; 50.9%)	Total (513; 100%)	
A. Tired					
Same or less	81 (71.7)	82 (59.0)	135 (51.7)	298 (58.1)	0.001
More	32 (28.3)	57 (41.0)	126 (48.3)	215 (41.9)	
B. Nervous					
Same or less	60 (53.1)	57 (41.0)	72 (27.6)	189 (36.8)	<0.001
More	53 (46.9)	82 (59.0)	189 (72.4)	324 (63.2)	
C. Anxious					
Same or less	58 (51.3)	46 (33.1)	60 (23.0)	164 (32.0)	<0.001
More	55 (48.7)	93 (66.9)	201 (77.0)	349 (68.0)	
D. Sad					
Same or less	76 (67.3)	69 (49.6)	102 (39.1)	247 (48.1)	<0.001
More	37 (32.7)	70 (50.4)	159 (60.9)	266 (51.9)	
E. Aggressive					
Same or less	77 (68.1)	82 (59.0)	114 (43.7)	273 (53.2)	<0.001
More	36 (31.9)	57 (41.0)	147 (56.3)	240 (46.8)	
F. Family conflicts					
Same or less	86 (76.1)	82 (59.0)	124 (47.5)	292 (56.9)	<0.001
More	27 (23.9)	57 (41.0)	137 (52.5)	221 (43.1)	

When evaluating the child’s age, regressive behaviors occurred more frequently in the age group of 3–6 years (57.1%) and between 7 and 10 years (44.6%), with a statistically significant association, such as described in Table 4.

**Table 4.** Association between the age and gender of the child and the occurrence of regressive behaviors.

Variable	Has your child been exhibiting age-inappropriate behaviors (e.g., bedwetting, going to bed with parents when he or she was not doing so, childish speech, increased dependence on parents, etc.)?			p-value
	No (n=331) (%)	Yes (n=211) (%)	Total (n=542) (%)	
How old is your child? (years)				
0–2	46 (68.7)	21 (31.3)	67 (100)	<0.001
3–6	79 (42.9)	105 (57.1)	184 (100)	
7–10	92 (55.4)	74 (44.6)	166 (100)	
11–17	114 (91.2)	11 (8.8)	125 (100)	
What is your child’s gender?				
Male	165 (49.8)	117 (55.5)	282 (52)	0.183
Female	156 (47.1)	87 (41.2)	243 (44.8)	
Not informed	10 (3)	7 (3.3)	17 (3.1)	

There was no association between the child’s gender and the occurrence of regressive behaviors (p=0.18).

## DISCUSSION

This study shows, during COVID-19 pandemic, a high rate of absenteeism from routine medical appointments and procedures (38%), excessive screen time (50.9%), and sedentary lifestyle (39.1%). Still, the participants reported an increase in the perception of nervousness (62.7%), anxiety (67.7%), and sadness (51.3%) during this period. Regressive behaviors occurred more frequently in the age group of 3–6 years (57.1%) and between 7 and 10 years (44.6%).

The COVID-19 pandemic impacted the health of the population in different ways. A Brazilian study reported a high prevalence of adult Brazilians with sleep problems, anxiety, nervousness, and sadness compared to the beginning of the pandemic.^6^ Such data reinforce what was obtained in this study. In our sample, it was observed that 81% of the responsible persons were more tired and 79.3% more anxious than in the period before the pandemic, and 43.3% of the responsible persons were reported to have great difficulty in sleeping.

Social isolation, besides causing psychological and physical symptoms, makes difficult the development of preschool and school children.^4^ This study shows that several children, especially in the aforementioned phases, aged between 3 and 10 years, showed regressive behaviors such as childish talk, nocturia, excessive fondness to parents, and going to bed with them when he or she was not doing so. Confinement in the home, inability to attend schools, isolation, and the presence of death as a frequent theme are triggering points for the decline in children’s intellectual and behavioral development. Furthermore, they are conditions that, by themselves, are in favor of the emergence of a child’s toxic stress.^2,4^ In this study, the age group that presented the most psychological symptoms was composed of children aged between 3 and 6 years.

A child’s toxic stress arises from multiple factors and social isolation, resulting from the COVID-19 pandemic. Exposure to dysfunctional family environments, with situations of domestic violence, constant conflicts, and constant fear, encourages the occurrence of toxic stress. In addition to these negative situations, the children’s physical vulnerability, emotional immaturity, and the inability to access help from other environments cause the pandemic to proliferate toxic childhood stress and lead to important psychological impairment.^4^ This commitment can be even greater for children who previously experienced domestic violence and sexual abuse, or faced psychological problems arising from family issues.

The concern also turns to children who faced the pandemic during their early childhood period, given the recognized importance of the first thousand days in their development.^4,7^ In this period, there is intense neural development, and multiple factors arising from the pandemic interfere in a harmful way in this process. Inadequate sleep and lack of stimulation due to reduced social contact contribute to language and communication limitation and directly affect social skills and behavior.^8^ Also, negatively impacting the development of those in social isolation, there are inadequate nutrition, sedentary lifestyle, long periods of loneliness, and the dysfunctional family environment.

In contrast, healthy family environments, which enable adequate stimulation and education, as well as enhanced positive parental relationships, seem to promote cognitive and psychological gains. Also, the encouragement of parents for proper eating habits, physical activities, and reduced exposure to screens allows to minimize the impacts of the pandemic on the development of children, highlighting that family influence can minimize or increase losses.^4^

Besides the great losses in the formal learning process, children and adolescents have been deprived of the necessary socialization with peers, where significant learning for human development takes place.^2^ In total, 75.5% of parents or responsible persons participating in this study answer that their children had activities of schools in a virtual way. Of the 497 children who attend schools in the study sample, 409 were unable to attend in person during the study period. Social isolation, complete or partial, was present in 85.6% of the answers and in these situations the virtual environment became an instrument for maintaining bonds and contacts. Thus, virtual conversations through electronic equipment seem to contribute to increased exposure to screens.^9^

The Brazilian Society of Pediatrics recommends that the exposure to screens by children aged under 2 years should be absolutely avoided and limited up to 1 h for children aged between 2 and 5 years, 2 h for the age group of 6 and 10 years, and 3 h for adolescents.^10^ It is known that there is a great number of losses associated with the excessive use of technologies in children and adolescents, with effects that can extend in the long term. We can mention digital dependence, anxiety disorders, depression, and irritability, in addition to exposure to situations of vulnerability such as violence, cyberbullying, pornography, grooming, sexting, among others.^11^

The increase in time of exposure to screens, observed in this study, highlights this problem, leading to a concern about its consequences in the post-pandemic period. In all, 50.9% of the participants reported exposure time to screens greater than 4 h a day, except for the time of participation in virtual classes, an average higher than that found in previous studies and higher than the recommendations for all age groups.^10,12^ This was evidenced with a positive correlation between screen time and the frequency of psychic symptoms and family conflicts.

During school holidays, when children and adolescents remain at home, without outdoor activities and without interaction with people of the same age, harmful effects on the health of this group are observed. Such effects include insomnia, irregular nutrition, weight gain, and reduced or absence of physical activity.^4,13^ These implications are also found in young people who face long periods of confinement, as occurred in the new coronavirus pandemic.^14^ Daily changes caused by the pandemic evidenced several psychological problems, such as pathological stress, depression, fear, feeling of loneliness, post-traumatic stress disorder (PTSD), and anxiety attacks.^4,15,16^ Children who have experienced traumatic situations, for example, have similar rates of PTSD as isolated children in quarantine.^1^

In terms of health care, there was a reduction in vaccination coverage and delay in childcare appointments.^17,18^ Delay in vaccinations can expose children to a higher risk of mortality from vaccine-preventable diseases.^18^ Absenteeism in pediatric appointments that was found in our study is relevant, with 38.0% of the participants reporting postponement or suspension of appointments or treatments during the period.

Furthermore, the practice of physical activities is an important health promoter and plays a key role in child development, but during the pandemic, sedentary lifestyle was exponentially enhanced. A study carried out in 2021 with young people aged 10–19 years, during the COVID-19 pandemic, showed a high level of physical inactivity, before and during confinement, aggravated by the lockdown measures of the studied countries.^7^ In this study, it was found that 39.1% of the children did not practice any type of physical activity. Still, among those who practiced it, 76.67% devoted less than 3 h a week to such activities, a value much lower than the 60 min a day recommended by the WHO for children and adolescents.^19^

Considering the limitations of this study, it was observed that the sample was composed of individuals with high education and income considerably higher than the average for the Southern Region and exponentially higher than the national average.^20,21^ This fact allows that the findings of this study do not correspond to the reality of other social classes or regions in Brazil. However, considering that poorer families suffer more from the pandemic, the impacts found in this study may be even bigger in more vulnerable populations.^22^ Another limitation is the possibility of the responsible persons having answered more than one questionnaire, when they have more than one child, which would impact the interpretation of data acquired about family structures.

In conclusion, this study demonstrated the great negative impact of the pandemic and social isolation among children and adolescents regardless of age group. The deleterious effects occurred in terms of both physical and mental health.

Given the findings of this study, it is evident that toxic stress is a real problem in the pediatric population during the pandemic. For that matter, it is necessary for parents, educators, health professionals, and everyone who lives with children and adolescents to watch out for any sign or symptom in order to interfere early and enable children and adolescents to enjoy full physical health, mental, and social.
